# CT characteristics of non-small cell lung cancer with epidermal growth factor receptor mutation: a systematic review and meta-analysis

**DOI:** 10.1186/s12880-016-0175-3

**Published:** 2017-01-10

**Authors:** Zenghui Cheng, Fei Shan, Yuesong Yang, Yuxin Shi, Zhiyong Zhang

**Affiliations:** 1 Department of Radiology, Shanghai Public Health Clinical Center, Fudan University, NO.2901 Caolang Road, Jinshan, Shanghai, 201508 China; 2 Department of Radiology, Qingpu branch of Zhongshan Hospital, Fudan University, Shanghai, 201700 China; 3 Department of Medical Imaging, Sunnybrook Health Sciences Center, University of Toronto, Toronto, ON M4N 3M5 Canada

**Keywords:** Computed tomography, EGFR, Non-small cell lung cancer

## Abstract

**Background:**

To systematically investigate the relationship between CT morphological features and the presence of epidermal growth factor receptor (EGFR) mutations in non-small cell lung cancer (NSCLC).

**Methods:**

All studies about the CT morphological features of NSCLC with EGFR mutations published between January 1, 2000 and March 15, 2015 were searched in the PubMed and EMBASE databases. Qualified studies were selected according to inclusion criteria. The frequency of EGFR mutations and CT features of ground-glass opacity (GGO) content, tumor size, cavitation, air-bronchogram, lobulation, and spiculation were extracted. The relationship between EGFR mutations and each of these CT features was tested based upon the weighted mean difference or inverse variance in the form of an odds ratio at a 95% confidence interval using Forest Plots. The publication bias was examined using Egger’s test.

**Results:**

A total of 13 studies, consisting of 2146 NSCLC patients, were included, and 51.12% (1097/2146) of patients had EGFR mutations. The EGFR mutations were present in NSCLC with part-solid GGO in contrast to nonsolid GGO (OR = 0.49, 95% CI = 0.25–0.96, *P* = 0.04). Other CT features such as tumor size, cavitation, air-bronchogram, lobulation and spiculation did not demonstrate statistically significant correlation with EGFR mutations individually (*P* = 0.91; 0.67; 0.12; 0.45; and 0.36, respectively). No publication bias among the selected studies was noted in this meta-analysis (Egger’s tests, *P* > 0.05 for all).

**Conclusion:**

This meta-analysis demonstrated that NSCLC with CT morphological features of part-solid GGO tended to be EGFR mutated, which might provide an important clue for the correct selection of patients treated with molecular targeted therapies.

**Electronic supplementary material:**

The online version of this article (doi:10.1186/s12880-016-0175-3) contains supplementary material, which is available to authorized users.

## Background

Lung cancer is the leading cause of cancer-related deaths globally, with an estimated 1,589,900 deaths in 2012 [1]. In the USA, over 220,000 patients with lung cancers were diagnosed in 2015, and the 5-year overall survival was only 18% [2]. In China, approximately 733,300 patients with lung cancers were diagnosed and 610,200 of them died in 2015; the number of deaths would be anticipated to be more than one million by 2025 [3, 4]. Most patients with lung cancer are diagnosed at advanced stages and are not eligible for curative surgery due to the lack of early specific signs and symptoms; hence, the prognoses for these patients are usually poor [5–7].

In recent years, the molecular targets of lung cancer, especially for the main histological type non-small cell lung cancer (NSCLC), have been investigated, including epidermal growth factor receptor (*EGFR*), Kirsten rat sarcoma viral oncogene homolog (*KRAS*), anaplastic lymphoma kinase (*ALK*), human epidermal growth factor receptor 2 (*HER2*), etc. Targeted therapy has shown promising benefits for patients who inherited mutations in these genes [8–13]. *EGFR*, one of these molecular targets with a high frequency of mutation, is a transmembrane receptor tyrosine kinase involved in the signaling pathways regulating cell proliferation, apoptosis, angiogenesis, and invasion [14, 15]. The most common *EGFR* mutations have been shown to be found in adenocarcinoma in female non-smoker of East Asian ethnicity [8, 9], and the mutation rate is reported to be 27–56% in this population compared with 8–10% worldwide [9, 16]. Patients with *EGFR* mutations demonstrated a high response rate of approximately 70% to EGFR tyrosine kinase inhibitor (EGFR-TKI) therapy. The progression-free survival (PFS) has been reported to reach 9 to 13 months when EGFR-TKIs are administered as the first-line therapy [17–19]. Two types of method for detecting *EGFR* mutations are currently available: “screening” assays that detect overall mutations, such as Next Generation Sequencing (NGS) and Sanger Sequencing, and “specific” methods that detect specific known mutations using different approaches, such as Roche’s *EGFR* Mutation Test and Life Technologies’ SNaPShot [20, 21]. However, both methods are costly and not feasible in every lung cancer clinic. CT is a routinely used and relatively cost-effective modality in the diagnosis of lung cancer that presents various imaging features, some of which have been reported to relate with certain histopathological types [22], while these types have been identified to correlate with *EGFR* mutations [23]. Therefore, we hypothesized that specific CT features of NSCLC were associated with *EGFR* mutations. In this study, we systematically searched the current medical literature and comprehensively examined the relationship between CT features and the presence of *EGFR* mutations in NSCLC patients.

## Methods

This meta-analysis was carried out in accordance to the Preferred Reporting Items for Systematic Reviews and Meta-analyses (PRISMA) statement (Additional file 1. Checklist S1) [24]. The primary procedures were as follows:

1. Search strategy
  We searched PubMed and EMBASE (Excerpta Medica database) for all articles about radiogenomics of NSCLC with EGFR mutation published between January 1, 2000 and March 15, 2015. The medical subject terms and key words used for search were “epidermal growth factor receptor”, “EGFR”, “lung cancer”, “lung carcinoma”, “CT”, and “imaging” in the Boolean expression: ((epidermal growth factor receptor) OR EGFR) AND ((lung cancer) OR (lung carcinoma)) AND ((CT) OR (imaging)) without language restrictions. Related articles, including those from the references, were also searched.
2. Inclusion/exclusion criteria
  Qualified studies were included if they satisfied the following criteria: (1) NSCLC was diagnosed based upon either pathological or cytological results; (2) EGFR mutations were determined by fluorescence in situ hybridization (FISH), immunohistochemistry (IHC), polymerase chain reaction (PCR), or any combination of the above-mentioned methods; (3) CT features of tumors were studied before the determination of EGFR mutation or afterwards in a blinded manner; (4) the association between EGFR mutation status and CT features was investigated; and (5) studies were available with full text articles. The studies were excluded if (1) there was duplicate data or insufficient data; and (2) the articles were abstracts, comments, narrative reviews, or editorials without full-text available.
3. Data extraction
  The following information was independently extracted from all eligible articles by two investigators (Cheng Z.H. and Shan F.): first author’s name, year of publication, country of origin, number of enrolled patients, frequency of the EGFR gene mutation, detection method, histologic type, and CT features, which included proportion of ground-glass opacity (GGO), tumor size, cavitation, air-bronchogram, lobulation, and spiculation. GGO was defined as hazy intensity with visible brochovascular markings in the lung window setting [25]. The proportion of GGO was calculated according to the ratio of the maximum length of GGO to that of total tumor in the largest cross section and classified as follows: (1) solid tumor: GGO = 0%; (2) part-solid GGO: 0% < GGO <50%, and 50% ≤ GGO <100%; (3) non-solid GGO = 100% [26–29]. Tumor size was measured in the largest cross section by averaging the length and width, and in the largest tumor if multiple tumors were present [26]. Cavitation was defined as airspace within the tumor at the time of diagnosis and prior to biopsy or treatment [30]. Air-bronchogram was defined as air-filled small foci or branches within the solid part of tumor [31]. Lobulation was defined as the shallow wavy contour of a tumor’s surface with exception of the portion adjacent to pleura [32]. Spiculation was defined as sharp linear projections from the tumor [31]. All the above features were analyzed for each tumor prior to treatment. Any discrepancies between the independent extractions of data were resolved by a mutual review of the original articles for a consensus agreement.
4. Statistical analysis
  All statistical analyses were performed using the Review Manager (RevMan, version 5.3.5) and STATA (version 12.0). All statistical tests were two-sided, and the significance level was set at 0.05.

The association between the CT features and EGFR mutations of NSCLC was assessed based upon the weighted mean difference (WMD) or inverse variance (IV) in the form of odds ratio (OR) [33] at a 95% confidence interval (95% CI). Specifically, (1) the association between GGO content (containing GGO or not; non-solid, part-solid or solid; proportion of GGO) and EGFR mutation (some subtypes when available were also included) was tested by WMD for overall effect. (2) Association between tumor size and EGFR mutation was tested by IV for overall effect. (3) Association between tumor cavitation, air-bronchogram, lobulation, spiculation and EGFR mutation was tested by WMD for overall effect.

Heterogeneity was examined by the Chi-square based Q test. Inconsistency index (I^2^) ranging from 0 to 100% was utilized to define the inter-trial variability due to heterogeneity rather than to sampling error within the study [34]. A random-effects model based on the Der Simonian and Laird method was adopted if I^2^ was above 50%, which indicated the presence of a significant heterogeneity; otherwise, a fixed-effect model based on the Mantel-Haenszel method was used if I^2^ was under 50%.

The publication bias was examined using Funnel plots and Egger’s tests. Deviation from the funnel-shaped distribution of eligible studies may indicate the presence of publication bias.

## Results

### Qualified studies

**Fig. 1:**
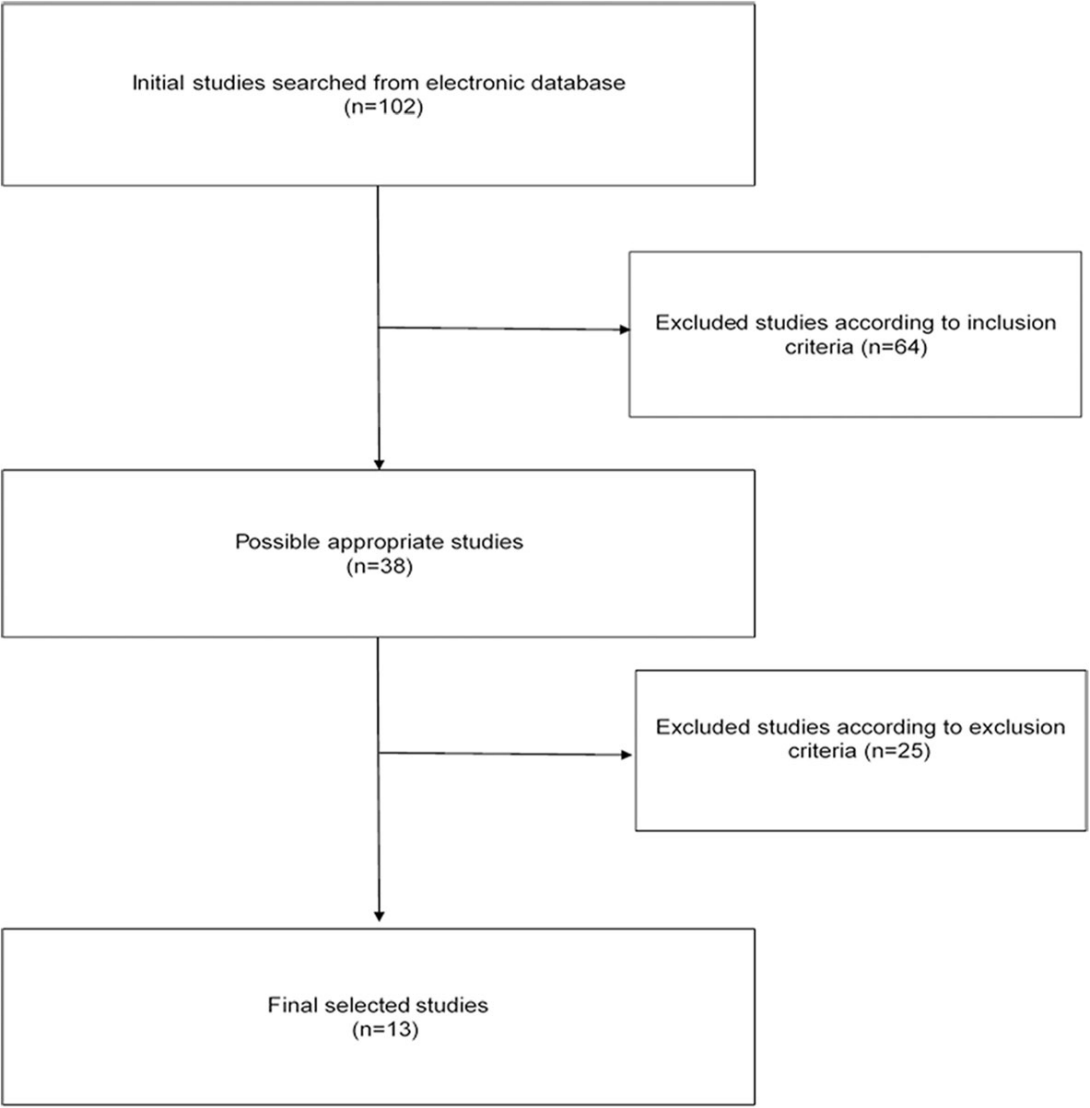
Flow diagram of the study selection process

A total of 2146 patients with NSCLC from 13 qualified articles were included [26–31, 35–41], of whom 1097 patients (51.12%) had EGFR mutations. Most studies (11/13) were from Asia, including Korea (3), Japan (5), and China (3); the other two studies were conducted in the U.S.A. The EGFR detection techniques included PCR (8 studies), FISH (1 study), IHC (2 studies), PCR and FISH (1 study), PCR and IHC (1 study). The flow diagram of the selection process of the eligible studies is shown in Fig. 1.

Table 1 summarizes the basic characteristics of all qualified articles. The rate of detection of EGFR mutations ranged from 23.83 to 73.91% based on 13 qualified articles, and the average incidence was 49.00% in 2146 patients with NSCLC.

**Table 1:** Summary of qualified studies

Study	Year	Source of Patient	Stage	No	Frequency	Method
Lee Y, et al.	2013	Korea	I	214	23.83% (51/214)	IHC
Lee HJ, et al.	2013	Korea	I–III	153	54.25% (83/153)	PCR, FISH
Park EA, et al.	2009	Korea	I–IV	132	40.15% (53/132)	FISH
Glynn C, et al.	2010	U.S.A	UN	64	32.81% (21/64)	PCR
Aoki T, et al.	2012	Japan	UN	25	40.00% (10/25)	PCR, IHC
Yano M, et al.	2006	Japan	I–III	80	47.50% (38/80)	PCR
Yoshida Y, et al.	2007	Japan	I	23	73.91% (17/23)	PCR
Hsu KH, et al.	2011	Taiwan	I	162	64.20% (104/162)	PCR
Sugano M, et al.	2011	Japan	I–III	136	41.18% (56/136)	PCR
Onn A, et al.	2005	U.S.A	I	72	66.67% (48/72)	IHC
Usuda K, et al.	2014	Japan	I–IV	148	39.19% (58/148)	PCR
Yang Y, et al.	2015	China	0–IV	788	60.91% (480/788)	PCR
Hsu JS, et al.	2014	Taiwan	III–IV	149	52.35% (78/149)	PCR

Notes: *UN* unknown, *IHC* immunohistochemistry, *PCR* polymerase chain reaction, *FISH* fluorescent in situ hybridization

### GGO and EGFR mutations

#### Tumors with or without GGO and EGFR mutation

**Fig. 2:**
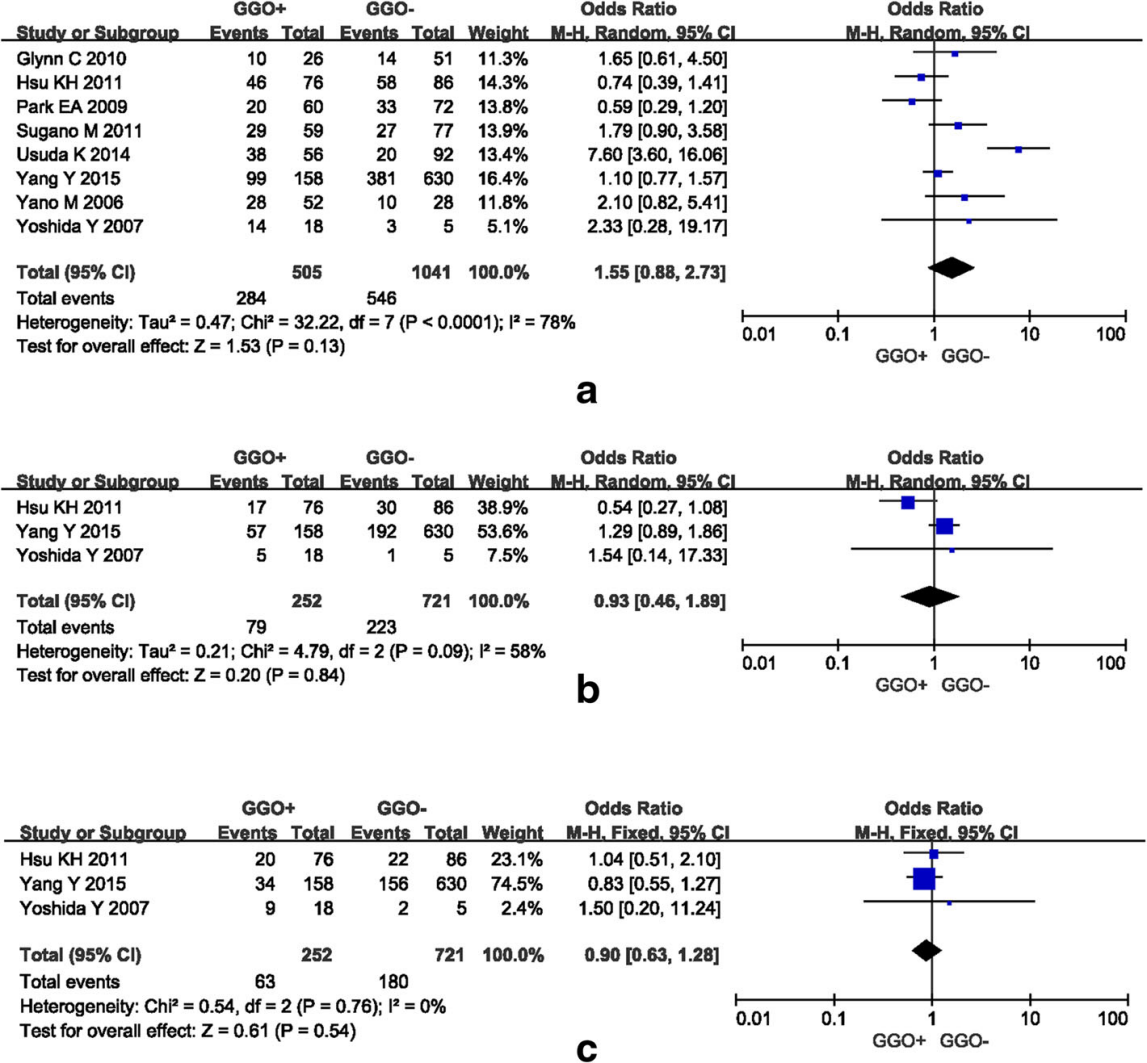
Forest plots of the studies comparing tumors with and without GGO and EGFR mutation (notes: events, tumors with EGFR mutation; total, all tumors with and without EGFR mutation). **a** No association was found between NSCLC with and without GGO content and overall EGFR mutation. **b** No association was found between NSCLC with and without GGO content and EGFR exon 21 mutation (L858R). **c** No association was found between NSCLC with and without GGO content and EGFR exon 19 deletion

Eight studies were available for investigation of the relationship between tumors with and without GGO and EGFR mutation. Out of a total of 505 tumors with GGOs and 1041 solid tumors (tumors without GGO), EGFR mutation was detected positively in 56.24% (284/505) and 52.45% (546/1041) of cases, respectively. Figure 2a summarizes the findings. A random-effects model was utilized for the meta-analysis due to significant heterogeneity (I^2^ = 78%, P < 0.0001). No statistically significant difference was found between tumors with and without GGO and EGFR mutation in patients with NSCLC (pooled OR = 1.55, 95% CI = 0.88–2.73, *P* = 0.13).

#### Tumors with or without GGO and EGFR mutation subtypes

Three studies were available for investigation of the relationship between tumors with and without GGO and the EGFR mutation subtypes, which included a total of 252 tumors with GGOs and 721 solid tumors. EGFR exon 21 mutation (L858R) was detected in 31.35% (79/252) and 30.93% (223/721) of cases, while EGFR exon 19 deletion was confirmed in 25% (63/252) and 24.97% (180/721) of cases, respectively. Figure 2b and c summarize the findings. A random-effect model and a fixed-effect model was chosen for meta-analysis as significant heterogeneity and no significant heterogeneity was observed, respectively (I^2^ = 58%, *P* = 0.09; I^2^ = 0%, *P* = 0.76). No statistically significant differences were found between tumors with and without GGO in patients with NSCLC having inherited these two mutation subtypes (OR = 0.93, 95% CI = 0.46-1.89, *P* = 0.84; OR = 0.90, 95% CI = 0.63–1.28, *P* = 0.54, respectively).

#### Non-solid GGO, part-solid GGO, solid tumor and EGFR mutation

**Fig. 3:**
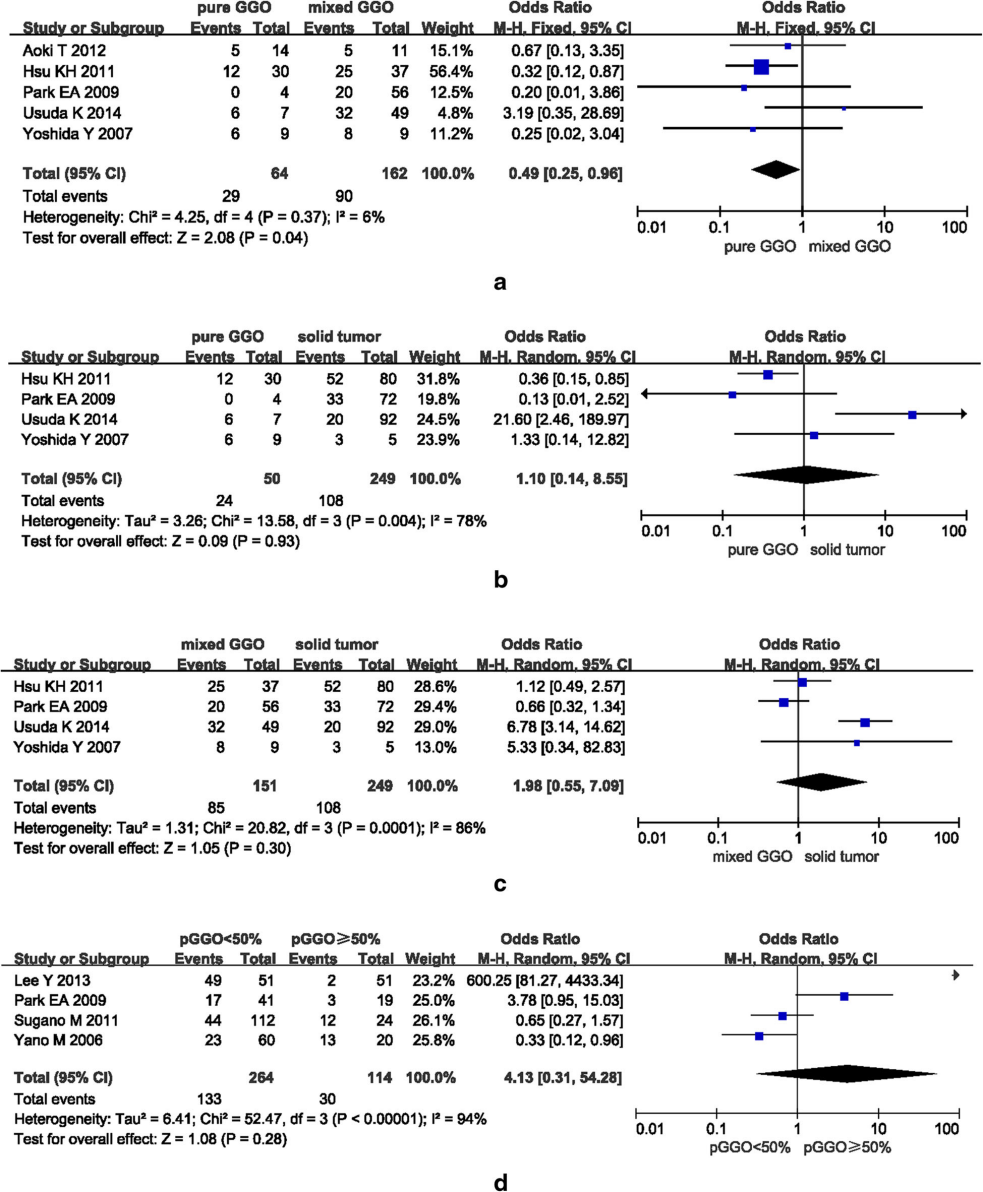
Forest plots of studies on the association between GGO volume and EGFR mutation. **a** Mixed GGO (part-solid GGO) was EGFR mutated much more commonly than pure GGO (non-solid GGO). **b**–**c** No association was found between pure or mixed GGO or solid tumor of NSCLC and EGFR mutation. **d** No association was found between GGO with percentages (pGGO) less than or greater than 50% of NSCLC and EGFR mutation

Five available articles were included for investigation of the relationship between non-solid and part-solid GGOs and EGFR mutation status. Out of a total of 64 tumors with non-solid GGOs and 162 part-solid GGOs, EGFR mutations were detected in 45.31% (29/64) and 61.73% (100/162) of cases, respectively. Figure 3a summarizes the findings. A fixed-effects model was used for meta-analysis as no significant heterogeneity was observed (I^2^ =6%, *P* = 0.37). The EGFR mutation rate was significantly higher in tumors with part-solid GGOs compared with pure ones (pooled OR = 0.49, 95% CI = 0.25–0.96, *P* = 0.04).

Four studies were available to study the relationship between non-solid GGOs and solid tumors or part-solid GGOs and solid tumors and the EGFR mutation status. A total of 50 non-solid GGOs, 151 part-solid GGOs and 249 solid tumors were found to have EGFR mutations in 48% (24/50), 56.29% (85/151), and 43.37% (108/249) of cases, respectively. Figure 3b and c summarize the findings. No statistically significant differences were observed between non-solid and solid tumors or part-solid and solid tumors and the EGFR mutation status, respectively (I^2^ = 78%, pooled OR = 1.10, 95% CI = 0.14–8.55, *P* = 0.93; I^2^ = 86%, pooled OR = 1.98, 95% CI = 0.55–7.09, *P* = 0.30, respectively).

#### Proportion of GGO and EGFR mutation

Four studies investigated the relationship between tumors with a proportion of GGO less than or no less than 50% and EGFR mutation. Of a total of 264 tumors in the former group and 114 in the latter, EGFR mutations were detected in 50.38% (133/264) and 26.32% (30/114) of cases, respectively. Figure 3d summarizes the findings. A random-effects model was utilized for meta-analysis due to significant heterogeneity (I^2^ = 94%, P < 0.00001). No statistically significant difference was found between these two groups and EGFR mutation (pooled OR = 4.13, 95% CI = 0.31–54.28, *P* = 0.28).

### Other morphological features and EGFR mutation

#### Tumor size and EGFR mutation

**Fig. 4:**
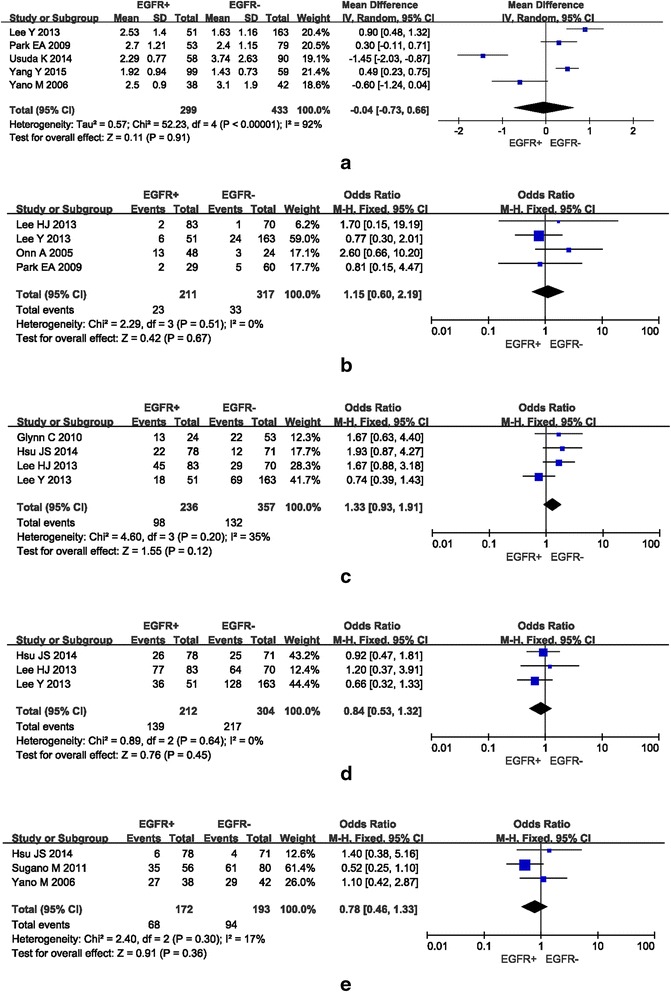
Forest plots of studies on the association between other morphological features of NSCLC and EGFR mutation. **a** No association was found between tumor size and EGFR mutation. **b**–**e** No association was found between other morphological features, such as cavitation, air-bronchogram, lobulation and spiculation, and EGFR mutation

Five studies investigated the relationship between tumor size and EGFR mutation status: a total of 299 NSCLCs with average size ranging from 1.92 to 2.7 cm with inherited EGFR mutation and 433 tumors measuring between 1.43 and 3.74 cm without EGFR mutation were pooled into the meta-analysis. Figure 4a summarizes the findings. A random-effects model was adopted because of significant heterogeneity (I^2^ = 92%, P < 0.00001). No statistically significant difference was demonstrated between NSCLCs with or without EGFR mutation and tumor size (pooled WMD = −0.04, 95% CI = −0.73–0.66, *P* = 0.91).

#### Tumor cavitation and EGFR mutation

Four studies investigated on the relationship between tumor cavitation and EGFR mutation status. A total of 23 out of 211 NSCLCs had cavitation with EGFR mutation (10.90%) compared to 33 out of 317 NSCLCs without EGFR mutation (10.41%). Figure 4b presents the findings. A fixed-effects model was adopted as no significant heterogeneity was noted (I^2^ = 0%, *P* = 0.51). No significant difference was observed between tumors with or without cavitation and EGFR mutation (pooled OR = 1.15, 95% CI = 0.60–2.19, *P* = 0.67).

#### Other CT features and EGFR mutation

In regard to relationship between tumors with or without air-bronchogram, lobulation, and spiculation and EGFR mutation, Fig. 4c, d, and e summarize the findings, respectively. The meta-analyses showed no significant differences between tumors with or without these CT features and EGFR mutation (*P* = 0.12, 0.45, and 0.36, respectively).

No publication bias was noted in this meta-analysis (Egger’s test, *P* > 0.05 for all). The summarized results are shown in Table 2.

**Table 2:** Summary of Egger’s tests

Meta-analysis	Egger's test	
	*t* value	*P* value
GGO (+/−) ~ EGFR	−0.11	0.915
GGO (≥/<50%) ~ EGFR	2.05	0.177
GGO (pure/mixed) ~ EGFR	0.71	0.551
GGO (mixed/-) ~ EGFR	0.22	0.847
GGO (pure/-) ~ EGFR	1.03	0.490
GGO (+/−) ~ exon21	−2.00	0.295
GGO (+/−) ~ exon19	7.70	0.082
Size ~ EGFR	−1.63	0.201
Cavitation (+/−) ~ EGFR	−1.89	0.199
Air-bronchogram (+/−) ~ EGFR	−0.31	0.787
Lobulation (+/−) ~ EGFR	0.50	0.703
Spiculation (+/−) ~ EGFR	−1.89	0.310

Notes: *GGO* ground glass opacity, *EGFR* epidermal growth factor receptor; + Indicating with; - indicating without; ≥/<50% indicating % GGO volume greater than/equal to or less than 50%

## Discussion

This meta-analysis investigated the radiogenomics of NSCLCs inherited with EGFR mutation and the results revealed that NSCLCs with part-solid GGOs rather than non-solid ones tended to be EGFR mutated. Other CT features such as tumor size, cavitation, air-bronchogram, lobulation and spiculation were not correlated with EGFR mutation.

The determination of the EGFR mutation status is crucial for the personalized treatment in patients with lung cancer and provides a molecular target that may be treated using anti-EGFR drugs. However, the successful detection of EGFR mutation is limited due to either insufficient pathological tissue collected by invasive aspiration or precluded due to the high cost of molecular examination. Therefore, a noninvasive and cost-effective modality is preferred. Based upon the reports that GGOs manifested on thin-section CT have been found to be associated with certain histopathological types, such as atypical adenomatous hyperplasia (AAH), adenocarcinoma in situ (AIS, previously known as bronchioloalveolar carcinoma (BAC)), and minimally invasive adenocarcinoma (MIA, previously known as adenocarcinoma with a predominant BAC component (ABAC)) [22], and that EGFR mutation is frequently detected in these pathological subtypes, we sought to study whether the detection of GGO on CT correlates with EGFR mutation.

Although many retrospective studies have reported that GGO was more frequent in tumors with EGFR mutation [28, 37, 39–41], this meta-analysis revealed that NSCLC with or without GGO did not differ in terms of their EGFR mutation status. A possible explanation may be the heterogeneity of the study population related to some demographic or clinical features. Just as Sugano M et al. [29] reported that there was no significant association between GGO and EGFR mutation, but that the EGFR mutation occurred more frequently in male patients with GGO, this gender difference may be accounted for by cross-talk between EGFR and estrogen receptors [42]. Such subgroup analysis was not performed in this meta-analysis because of the lack of qualified studies available. Additionally, the two most common activating mutation subtypes, short in-frame deletions of exon 19, and point mutation (CTG to CGG) in exon 21 at nucleotide 2573 (L858R) [43], did not differ in NSCLCs with or without GGO neither, although Hsu et al. [36] found that a typical EGFR mutation, especially L858R, was more frequent in tumors (stage I) with invasive solid pattern and significantly less in tumors (stage I) with non-solid GGO. Again, the heterogeneity in different tumor stages or histological subtypes may have played a role in this aspect.

Regarding the proportion of GGO, this meta-analysis demonstrated that NSCLCs with part-solid GGO rather than the non-solid GGO tended to be EGFR mutated, which is consistent with results of several other recent studies indicating that mixed GGOs (part-solid GGOs), especially those with a lower percentage of GGO had a higher rate of EGFR TK domain mutation [27, 36, 41, 44]. A possible mechanism might be that EGFR, an oncogene, played an important role in carcinogenesis and tumor progression via activation of the RAS/RAF/MEK/MAPK and the PI3K/AKT/mTOR pathways if mutated [45, 46] and that the incidence of EGFR mutation may be up-regulated by enhanced activation of certain pathways during the progression of tumors from a non-solid GGO to a part-solid pattern.

In regard to the correlation between tumor size and EGFR mutation status, a tendency was found that the bigger the tumor was, the more frequent the EGFR mutated [26, 28, 38, 40], although the present meta-analysis did not show a statistically significant difference. This was probably due to other potential confounders that may have interacted with the tumor size. As Yano M et al. [28] noted, GGO was more frequently observed in EGFR mutation, and although a significant difference was not reached individually, there was a significant difference if taking both tumor size and proportion of GGO into consideration. Other CT features such as cavitation, air-bronchogram, lobulation and spiculation were also examined in this meta-analysis; however, no correlation with EGFR mutation was found. With more accumulated data in the future, a meta-regression may be utilized to further investigate the underlying interactive features.

There are some limitations in this meta-analysis. First, the sample size in a few subgroups was small, thus the test effect may be lower and a false negative finding would be introduced. Second, as there were no randomized controlled trials (RCT) available and the majority of studies were retrospective, this may have introduced a selection bias that could influence the final overall effect. Third, CT scanning parameters and EGFR mutation detection methods were heterogeneous across the retrieved studies, and this may have increased the risk of inter-study heterogeneity. Lastly, meta-regression analysis was not performed due to the small number of subgroups.

## Conclusions

In conclusion, this meta-analysis demonstrated that EGFR mutation tended to be inherited in NSCLCs with part-solid GGOs compared tumors with non-solid GGO pattern. There was no correlation between EGFR mutation and other CT features such as tumor size, cavitation, air-bronchogram, lobulation and spiculation. As most eligible studies were retrospectively performed and had a relatively small sample size, future prospective studies with a larger sample size are warranted for further clarification of the relationship between molecular markers and CT morphological characteristics, thus providing supporting evidence for potential molecular targets that may be treated using molecular drugs.

## Additional file

Additional file 1: PRISMA-checklist. (DOC 67 kb)

## Abbreviations

AAH: Atypical adenomatous hyperplasia
ABAC: Adenocarcinoma with a predominant BAC component
AIS: Adenocarcinoma in situ
ALK: Anaplastic lymphoma kinase
BAC: Bronchioloalveolar carcinoma
CT: Computed tomography
EGFR: Epidermal growth factor receptor
EGFR-TKI: EGFR tyrosine kinase inhibitor
FISH: Fluorescence in situ hybridization
GGO: Ground-glass opacity
HER2: Human epidermal growth factor receptor 2
I^2^: Inconsistency index
IHC: Immunohistochemistry
KRAS: Kirsten rat sarcoma viral oncogene homolog
MIA: Minimally invasive adenocarcinoma
NGS: Next generation sequencing
NSCLC: Non-small cell lung cancer
PCR: Polymerase chain reaction
PFS: Progression-free survival
PRISMA: Preferred Reporting Items for Systematic Reviews and Meta-analyses
RCT: Randomized controlled trials
